# Batch correction of single-cell sequencing data via an autoencoder architecture

**DOI:** 10.1093/bioadv/vbad186

**Published:** 2023-12-28

**Authors:** Reut Danino, Iftach Nachman, Roded Sharan

**Affiliations:** Blavatnik School of Computer Science, Tel Aviv University, Tel Aviv 6997801, Israel; School of Neurobiology, Biochemistry and Biophysics, George S. Wise Faculty of Life Sciences, Tel Aviv University, Tel Aviv 6997801, Israel; Blavatnik School of Computer Science, Tel Aviv University, Tel Aviv 6997801, Israel

## Abstract

**Motivation:**

Technical differences between gene expression sequencing experiments can cause variations in the data in the form of batch effect biases. These do not represent true biological variations between samples and can lead to false conclusions or hinder the ability to integrate multiple datasets. Since there is a growing need for the joint analysis of single**-**cell sequencing datasets from different sources, there is also a need to correct the resulting batch effects while maintaining the true biological variations in the data.

**Results:**

We developed a semi-supervised deep learning architecture called Autoencoder-based Batch Correction (ABC) for integrating single**-**cell sequencing datasets. Our method removes batch effects through a guided process of data compression using supervised cell type classifier branches for biological signal retention. It aligns the different batches using an adversarial training approach. We comprehensively evaluate the performance of our method using four single**-**cell sequencing datasets and multiple measures for batch effect removal and biological variation conservation. ABC outperforms 10 state-of-the-art methods for this task including Seurat, scGen, ComBat, scanorama, scVI, scANVI, AutoClass, Harmony, scDREAMER, and CLEAR, correcting various types of batch effects while preserving intricate biological variations.

## 1 Introduction

The decreasing cost of sequencing techniques, as well as the important insights achieved by the analysis of gene expression data, have led to the generation of enormous single**-**cell RNA sequencing atlas projects, such as the Human Cell Atlas (Rozenblatt-Rosen *et al.* 2021), which contain datasets generated by multiple laboratories under different settings. These different conditions introduce batch effects into the data in the form of noise in varying distributions and intensities that can lead to false conclusions if not corrected.

Previous integration methods fall into various categories, depending on their underlying assumptions, algorithmic or architectural types, or their incorporation of known biological variations, often represented by cell type annotations. An example of this is scVI (Lopez *et al.* 2018) and its extension scANVI (Xu *et al.* 2021) for annotated data, which assume a zero-inflated negative binomial distribution of expression data across batches. Another example is ComBat (Johnson *et al.* 2006), which assumes Gaussian distributions. However, gene expression data have been shown to follow diverse distribution types (de Torrenté *et al.* 2020), and these singular assumptions can potentially lead to misinterpretations of the data (Svensson 2020).

Moreover, integration methods that overlook known biological differences between cells, particularly in the context of varying batch effects on different cell types, risk eliminating biological signals as part of batch effect noise. For instance, anchor-based methods like Seurat (Stuart *et al.* 2019) and Scanorama (Hie *et al.* 2019) estimate batch effects by identifying mutual nearest neighbor cells across batches in a dimensionally reduced data space, applying local correction vectors to each cell. Another example is Harmony (Ilya *et al.* 2019), which operates by projecting the data to its principal component (PC) space and employing k-means clustering in this space. It evaluates and adjusts for the influence of batch identity on cell PC coordinates, iteratively shifting cells toward the centroid of their designated cluster. This process, repeated until convergence, aligns cells across different batches, effectively reducing batch-specific variations. However, these methods do not utilize prior biological knowledge, such as cell type labels, in anchor computation or PC space clustering, leading to potential over-correction. Similarly, ComBat and scVI, which also disregard biological variation in their correction algorithms, are also prone to this over-correction.

Recent years have seen the development of deep learning methods for batch effect correction, many utilizing an autoencoder architecture (Bank *et al.* 2023), including scVI and scANVI. For instance, scGen (Lotfollahi *et al.* 2019) which is a perturbation modeling tool used to predict gene expression response to perturbation can also be used for batch correction. It uses a variational autoencoder architecture (VAE) and latent space arithmetic to linearly extrapolate gene expression from cells of one batch using another one in latent space and infer corrected non-linear predictions of gene expression vectors in feature space. This method uses cell type labels to account for biological variations in the data. In our comparison, we found it to outperform the previously mentioned methods. AutoClass (Li *et al.* 2022), another autoencoder-based architecture, includes a cell type classifier in its latent space to preserve biological variance and guide the encoding stage, similar to our approach. However, the decoding stage of this method remains unguided. scDREAMER (Ajita *et al.* 2023) is another VAE-based method, which also includes a batch classifier and a discriminator. The batch classifier is adversarially trained alongside the encoder, to accurately predict and adjust for batch information. The discriminator’s role is to distinguish between the original and the reconstructed expression profiles, aiding in the refinement of the autoencoder’s reconstruction capabilities. However, the reconstructed data are decoded in an unsupervised manner, not utilizing known cell type annotations, which could potentially limit its ability to conserve important biological variations. Another deep learning integration method, CLEAR (Han *et al.* 2022), is based on self-supervised contrastive learning, mapping single-cell RNA-seq data to a low-dimensional space using an encoder network. It incorporates simulated noise, including Gaussian noise and dropout events, into gene expression profiles. This creates positive pairs (original and distorted profiles from the same cell) and negative pairs (profiles from different cells). During training, the model produces similar representations for positive pairs and distinct ones for negative pairs, aligning functionally similar cells in the transformed space. However, this approach might suffer from limited generalizability if the simulated noise, meant to mimic batch effects, does not encompass all real-world data variations.

In this work, we developed Autoencoder-based Batch Correction (ABC), a deep learning architecture for the integration of single**-**cell sequencing data from multiple sources that removes batch effects while preserving the biological variations in the data. Our method does not assume a known distribution of the data nor the presence of the same cell types across all batches. Rather, ABC is based on an autoencoder architecture trained in an adversarial manner alongside a batch label discriminator, similar to Generative adversarial networks (Goodfellow *et al.* 2014). The architecture takes as input molecular measurements (e.g. mRNA levels or ATAC-seq levels) from a given cell, containing the normalized counts of each locus/gene in the cell, and outputs a corrected vector of values that can be used for downstream analysis. In our approach, cell type classifiers are utilized to guide both encoding and decoding processes, ensuring the retention of cell type-specific variations. This is particularly relevant for cell types that are unique to a specific batch and represented by a small number of cells. Moreover, we incorporate a multi-class batch classifier as a discriminator, during the adversarial training of the autoencoder, which is also informed by cell type information. This integration allows for a more nuanced, cell type-aware batch alignment, thereby improving the balance between mitigating batch effects and preserving biological variance. We evaluated our method on four multiple-batch datasets in its ability to remove batch effects and to conserve true biological variation following a previous comprehensive benchmark (Luecken *et al.* 2022). We compared our results to 10 previous state-of-the-art integration methods and demonstrate its distinct capability to integrate multi-species datasets, effectively preserving biological variance, particularly in terms of cell types and developmental trajectories, and to maintain spatial information in cell types whose functions are inherently dependent on their location.

## 2 Methods

### 2.1 The model and training process

**Figure 1.**
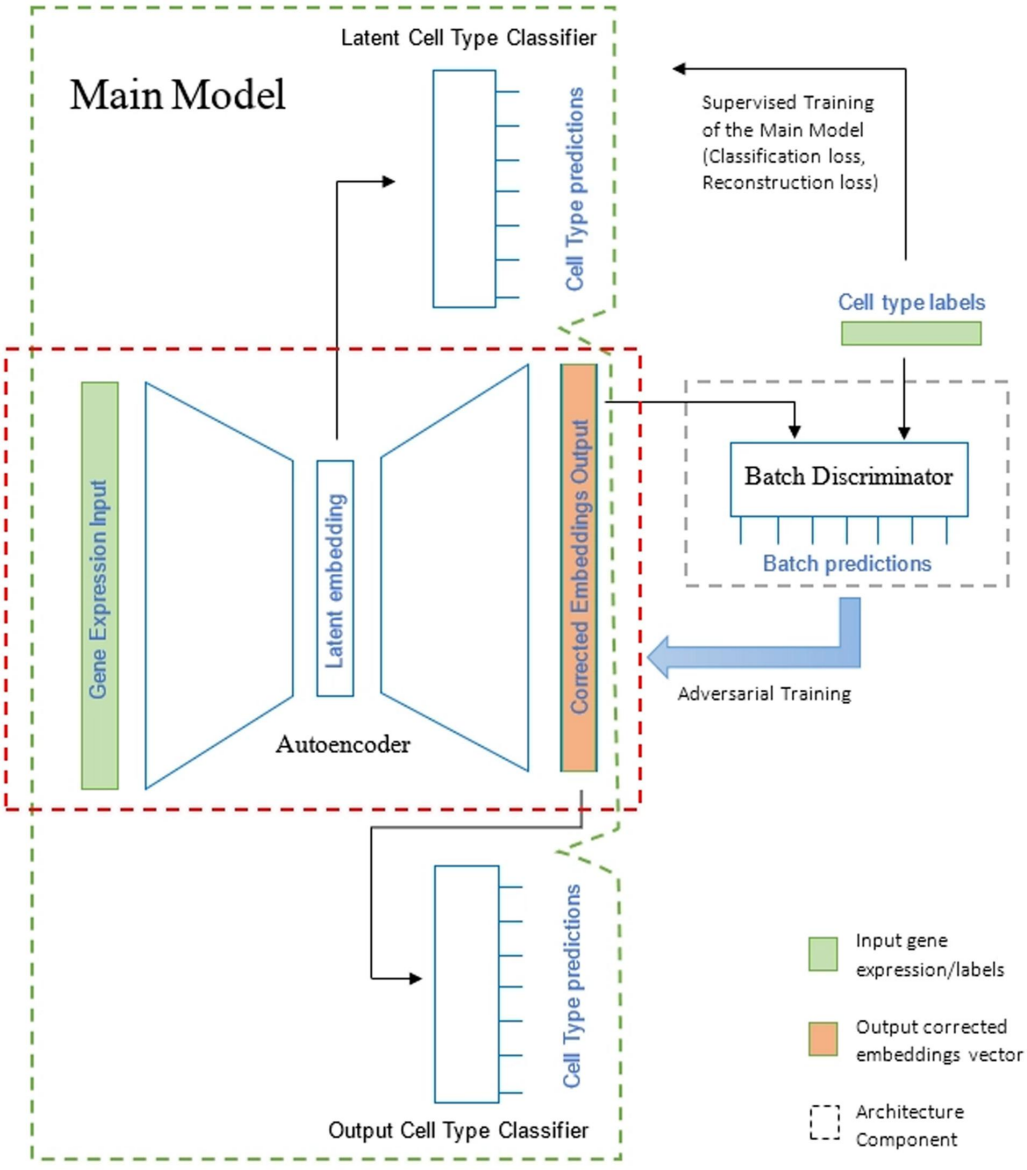
Full model architecture. The architecture is divided into three main components: the autoencoder, the main model, which includes the autoencoder layers and two cell type classifiers (the latent cell type classifier and the output cell type classifier) and a batch discriminator component, which is a batch label classifier used to train the autoencoder in an adversarial manner. The architecture receives as input a gene expression vector from a given cell and the cell’s type and batch (origin) labels, and outputs the corrected embedding.

ABC is based on an autoencoder architecture trained in an adversarial manner alongside a batch label discriminator to integrate sequencing datasets and remove batch effects while preserving the biological variation in the data. It receives as input a gene expression vector from a given cell, containing the normalized gene counts, and the cell’s type and batch (origin) labels, and outputs the corrected embedding. The architecture is depicted in Fig. 1. It consists of the *main model*, which includes an encoder (*E*), a decoder (*DC*), a latent cell type classifier (*LC*), and an output cell type classifier (*OC*) and is trained in an adversarial manner using a *batch discriminator* component (*DI*). All these sub-models use the *L*2 regularization to prevent overfitting and *ReLU* (rectified linear unit) activation function in their hidden layers. The encoder uses one dense hidden layer, and its output dense layer is the latent space layer used as input to the decoder and latent cell type classifier. Except for the output cell type classifier and the discriminator, which uses two dense hidden layers, all other sub-models use only one dense hidden layer. All classifiers use the *Softmax* activation in their output layer and the decoder uses the *Linear* activation function. The encoder uses *ReLU* activation in its output layer and also uses a dropout layer to prevent overfitting. The loss function used by the classifiers and discriminator is the (regularized) categorical cross-entropy, which is calculated between the predicted vector of probabilities and the ground truth label, while the autoencoder (encoder and decoder) uses a (regularized) mean squared error loss as a reconstruction loss. The training process is divided into three stages, in each one the weights of a different component are updated through gradient descent.

First, the discriminator is trained to predict the given cell’s batch label out of *C* batches in total. Denote by *x* the input expression matrix (where $x_{i}$ is the expression vector for cell *i*), $\bar{x}$ the corrected matrix, E(x) the latent (encoded) representation, *tl* a cell-type labeling matrix (where $tl_{i}$ is a one-hot encoded labeling vector for cell *i*), and *bl* a batch labeling matrix. The discriminator’s loss is an average over all *n* cells in the training set of a cross-entropy measure that is regularized by the $L_{2}$ norm of the model’s parameters $W^{DI}$:

(1) $$\begin{array}{c} {\text{CCE}}_{\text{DI}}(\text{DI}(\bar{x},tl),bl)=\frac{1}{n}\sum_{i=1}^{n}\sum_{c=1}^{C}bl_{i,c}\;\text{log}({\text{DI}}_{l}{\left({\bar{x}}_{i},tl_{i}\right)}_{c})\\ +\;\lambda \sum \left|\right|W^{DI}|{|}^{2} \end{array}.$$

In the second stage, the autoencoder is trained, updating the weights of both the encoder and decoder sub-models. This training is done in an adversarial manner, opposing the discriminator, and its loss function depends entirely on the discriminator’s output: the probability of the correct batch (denoted *c* in the equation) is used as the error estimation for a cross-entropy loss.

(2) $$L_{\text{ADV}}=-\frac{1}{n}\sum_{i=1}^{n}\;\text{log}\;(1\;-\;\text{DI}{\left({\bar{x}}_{i},tl_{i}\right)}_{c}).$$

The last stage of the training process updates all model’s weights, apart from the discriminator, by using a weighted loss function combining the loss functions of the cell type classifiers and the autoencoder reconstruction loss:

(3) $$\begin{array}{c} L:=\lambda_{1}\text{MSE}(x,\bar{x})\;+\;\lambda_{2}\text{CCE}(\text{LC}(E(x)),tl)\\ \;+\;\lambda_{3}\text{CCE}(\text{OC}(\bar{x}),tl) \end{array}.$$

All the components are trained using the Adam optimizer (Kingma and Ba 2014), while the learning rates, the latent space dimension and the weights in the main model’s loss are all hyperparameters we tune through a hyperparameter optimization process.

### 2.2 Hyperparameter tuning

We assessed the effect of the model’s hyperparameters using two datasets: an ATAC-seq dataset (Small mouse brain—Gene Activity) and an RNA-seq dataset (Human Pancreas) which were reserved for parameter tuning and were not included in the performance evaluation below. For the learning rate, we tested three popular values: 0.001, 0.0001, 0.0002 and a non-uniform approach that sets a different learning rate for the discriminator (0.001) as opposed to all other components (0.0001). We also tested different latent space dimensions: 32, 64, 128, 256, 512, 1024, 2048, and four weights for the reconstruction loss ($\lambda_{1}$) of the main model: 0.2, 0.4, 0.6, 0.8, setting the weights of the cell type classifiers to be $\lambda_{2}=\lambda_{3}=(1-\lambda_{1})/2$. The weights of the cell type classifiers are set to be equal to reduce the number of hyperparameters and since they share the same goal of conserving the biological signal. We trained our model for 50 epochs with all combinations of these values, and sorted the different combinations according to the final score of the integration they performed (Table 1). Finally, we chose the combination of hyperparameters that maximizes the average final score of the two datasets. The resulting hyperparameters values were a non-uniform learning rate, latent space dimension of 64, and a reconstruction loss weight of 0.8.

**Table 1.** Hyperparameter tuning results.

Parameters combination			Datasets scores (metrics averages)		Categories scores		
Learning rate	Latent dimension	Reconstruction loss weight	Mouse brain gene act. (ATAC)	Human pancreas (RNA)	Batch correction	Biological cons.	Average (Final score)
Non uniform	64	0.8	0.84286	0.70837	0.74636	0.80488	0.77562
Non uniform	256	0.4	0.8029	0.73697	0.6911	0.84877	0.76994
Non uniform	512	0.8	0.86629	0.7031	0.72475	0.81464	0.7697
0.001	512	0.4	0.82345	0.71496	0.71533	0.82308	0.76921
Non uniform	256	0.8	0.82588	0.70542	0.71652	0.81478	0.76565

### 2.3 Evaluation metrics

#### 2.3.1 Numerical scoring

The integration task was evaluated according to two different types of metrics, based on the two different goals of an integrating method: batch effect removal metrics which are used to evaluate how well samples from different batches are mixed, and biological variance conservation metrics that measures a method’s ability to conserve different biological signals in the data. When integrating scRNA-seq or scATAC-seq datasets, there is a trade-off between these two goals. All evaluations were performed according to a recently published integration benchmarking pipeline and using the package they developed (Luecken *et al.* 2022).

In short, batch and cell-type labels were used to evaluate the integration by comparing the clustering of cells before and after integration. When a strong batch effect is present in the data, the cells cluster according to their batch origin with minimal mixing between batches. After batch effects are removed, cells from different batches are expected to mix and only cluster according to the biological data (the different cell types), representing the conservation of the biological variance.

Three batch effect removal metrics were used: Average silhouette width (ASW) (batch), Graph Connectivity, and Graph iLISI. In addition, eight biological variance conservation metrics were used, divided into two groups—label dependent metrics: Normalized Mutual Information (NMI), Adjusted Rand index (ARI), ASW (cell-type), Graph cLISI, Isolated label F1, Isolated label silhouette, and label free metrics: cell cycle conservation and trajectory conservation (Luecken *et al.* 2022). We averaged the batch correction scores and the biological conservation scores and set the final integration score as the average of these scores:

(4) $$\begin{array}{c} \text{FinalScore}=0.5(\text{BatchCorrectionAvg})\\ +\;0.5(\text{BioConservationAvg}). \end{array}$$

In brief, the following measures were used: ARI and NMI compare the correspondence between cell-type labels and Louvain clusters calculated on the integrated dataset. ASW measures the relationship between the distance of a cell to cells within the same cluster and the distances of that cell to the closest cluster. Graph connectivity assesses whether the kNN graph of the integrated dataset connects all cells with the same cell type label directly. Graph LISI uses the inverse Simpson’s index to determine the number of cells *x* that can be drawn from a neighbor list before one batch is observed twice. cLISI and iLISI are than calculated using *x*, where cLISI is $\frac{B-x}{B-1}$ for *B* batches, and iLISI is the inverse. Isolated label scores evaluate how well the integration deals with cell type labels shared by few batches. They focus on labels which are present in the least number of batches and evaluate their separation from other cell types. Cell cycle conservation score evaluates how well the cell-cycle effect can be captured before and after integration. It computes scores for the cell cycle S and G2/M phases using Scanpy’s score_cell_cycle function (Wolf *et al.* 2018) and measures the difference between the variance contribution of the S and G2/M phase scores to PCs using PC regression. Trajectory conservation score computes Spearman’s rank correlation coefficient, between the pseudotime values before and after integration, which are calculated using diffusion pseudotime (*sc*.*tl*.*dpt* Scanpy method). All metrics are described in detail in Luecken *et al.* (2022).

#### 2.3.2 Trajectory conservation in multi species integration

We evaluated the conservation of trajectories in the multi-species Human And Mouse Immune Cells dataset, derived from bone marrow and peripheral blood cells, focusing on hematopoietic differentiation trajectory. Following integration, we employed Scanpy’s *scanpy*.*tl*.*diffmap* and *scanpy*.*tl*.*dpt* functions (Wolf *et al.* 2018) to calculate pseudotime and infer trajectories of selected cell types. We then demonstrated the alignment of our results with known hematopoietic differentiation process (see Section 3).

#### 2.3.3 Conservation of spatial states

We utilized the Lung Atlas dataset, which comprises cells from two distinct spatial locations: the airways and lung parenchyma. These different sampling locations inherently influence the cellular function and, consequently, the transcriptomic profiles of certain cell types. Specifically, cell types such as “Ciliated,” “Endothelium,” and “Secretory” are known to exhibit functional variations depending on their spatial context. Following integration, we first demonstrate ABC’s ability to conserve spatial information by plotting the integrated dataset on UMAP plots (McInnes *et al*. 2018), colored by cell type and location annotations, and identifying new sub-clusters aligning well with spatial information (see Section 3). Then, we perform sub-clustering within these major cell types, using *scanpy*.*tl*.*leiden* function (resolution = 0.25) (Wolf *et al.* 2018) to uncover finer cellular heterogeneity. Differential expression analysis was conducted for each identified sub-cluster using *scanpy*.*tl*.*rank_genes_groups*, allowing us to focus on the top differentially expressed genes and revealing distinct sub-populations informed by their origin (see Section 3), reinforcing our method’s capability to conserve spatial information.

### 2.4 Datasets and preprocessing

To evaluate our method, we performed the integration task on two scRNA-seq datasets and two scATAC-seq datasets (different from the two datasets used for hyperparameter tuning), taken from Luecken *et al.* (2022). We used the datasets: Human And Mouse Immune Cells, Human Lung Atlas, Small mouse brain (ATAC) Peaks, and Small mouse brain (ATAC) Windows. Each dataset contains data from multiple sources (batches) derived from different experiments, each of which consists of multiple cell types (Table 2). All datasets were quality controlled, normalized and log + 1 transformed. Finally, the top 3000 highly variable genes were selected for each dataset and used as the input.

**Table 2.** Datasets statistics.

Dataset	Cells	Batches	Types
Human And Mouse Immune Cells	97 861	23	19
Human Lung Atlas	32 472	16	17
Human pancreas (used for optimization)	16 382	9	14
Small mouse brain (ATAC) gene act. (used for optimization)	11 270	3	7
Small mouse brain (ATAC) Peaks	11 597	3	7
Small mouse brain (ATAC) Windows	10 761	3	7

**Table 3.** Runtime comparison.

Dataset size	scvi	scanvi	Scano-rama	combat	Seurat	Auto-class	scgen	Harmony	scDREA-MER	CLEAR	ABC	Avg.
10 761	322.1	352.6	30.0	4.5	147.9	93.4	80.9	7.1	370.0	975.0	139.3	229.3
11 597	348.7	376.9	35.2	4.7	135.1	101.1	74.1	8.0	Failed	994.0	147.0	222.5
32 472	590.3	668.0	157.1	11.6	2115.7	270.1	215.9	34.9	978.1	2823.0	386.2	750.1
97 861	582.4	865.0	503.2	47.5	12 845.2	782.4	629.9	72.4	3154.4	8652.0	1131.7	2660.5

## 3 Results

ABC is a semi-supervised deep learning architecture based on an autoencoder which integrates single**-**cell sequencing datasets and removes batch effects through a guided process of data compression, using supervised cell type classifier branches for biological signal retention and an unsupervised adversarial training for batch alignment. It consists of a Main Model, which includes an encoder, a decoder, a latent cell type classifier, and an output cell type classifier and is trained in an adversarial manner using a batch discriminator component. The architecture takes as input single**-**cell molecular measurements (mRNA levels or ATAC-seq levels) from a given cell, containing the normalized counts of each locus/gene in the cell, and outputs a corrected vector of values that can be used for downstream analysis.

We evaluated our method’s integration performance using four single**-**cell sequencing datasets with multiple measures for batch effect removal and biological variation conservation. The resulting integrated datasets demonstrate a clear separation between the different cell types and a very good mixture of the different batches, representing our methods ability to correct batch effects while maintaining the biological variance in the data. The results are presented first as the UMAP projections (McInnes *et al*. 2018) of the integrated datasets for clear visualization of the results and then in a metrics-based comparison between our method and 10 other integration methods. Finally, we demonstrate ABC’s ability to conserve spatial information in the integrated Lung Atlas and to conserve trajectories between species in the integrated Human And Mouse Immune Cells Atlas.

### 3.1 Dataset integration

**Figure 2.**
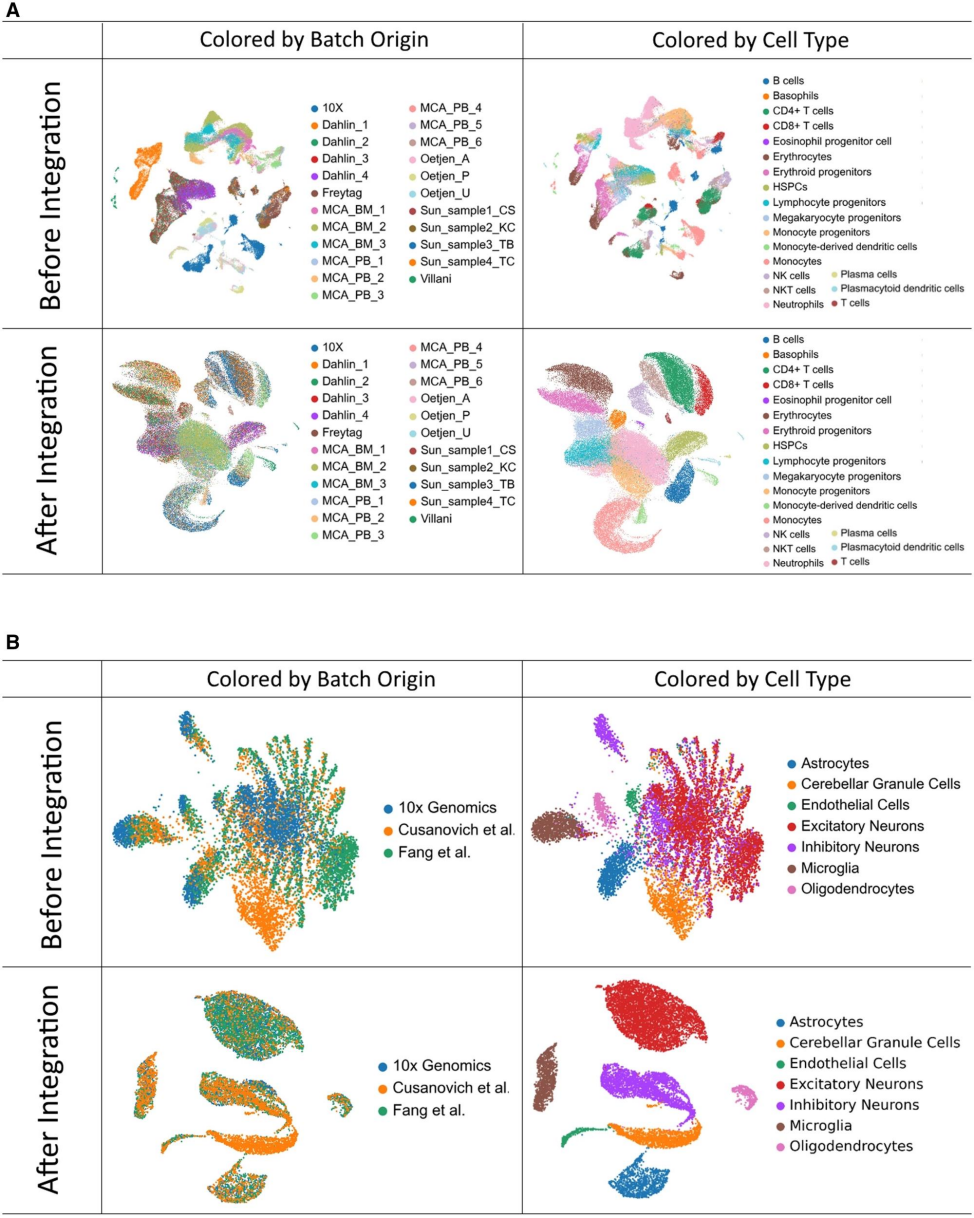
Before and after integration by ABC. (A) Human And Mouse Immune Cells Atlas projected on a UMAP, before and after integration by ABC. The dataset contains 97 861 cells from 23 different sources (batches) and 19 cell types. (B) Small mouse brain (ATAC) Peaks dataset projected on a UMAP, before and after integration by ABC. This dataset contains 11 597 cells from three different sources (batches) and seven cell types.

Our method’s ability to correct batch effects while maintaining the biological signal is demonstrated first by the UMAP projections (McInnes *et al.* 2018) of the datasets, before and after integration. All integrated datasets display distinct and separated cell type clusters, comprised of well-mixed batch origins. For example, the Human And Mouse Immune Cells Atlas dataset, the largest of our test datasets, poses the difficult challenge of integrating cells from multiple species. Nevertheless, ABC was able to integrate all 23 batches while conserving all 19 cell type identities, as demonstrated by the clear separation of cell type clusters in its UMAP projection (Fig. 2A). Similarly, the integrated Small mouse brain (ATAC) Peaks dataset, also displays well-mixed batches while forming distinct cell type clusters in its UMAP projection, even in cases where a certain cell type (Cerebellar Granule Cells) is uniquely present in only one of the batches (Fig. 2B). Results from additional datasets can be found in Supplementary Figs S1–S4.

### 3.2 Performance evaluation

**Figure 3.**
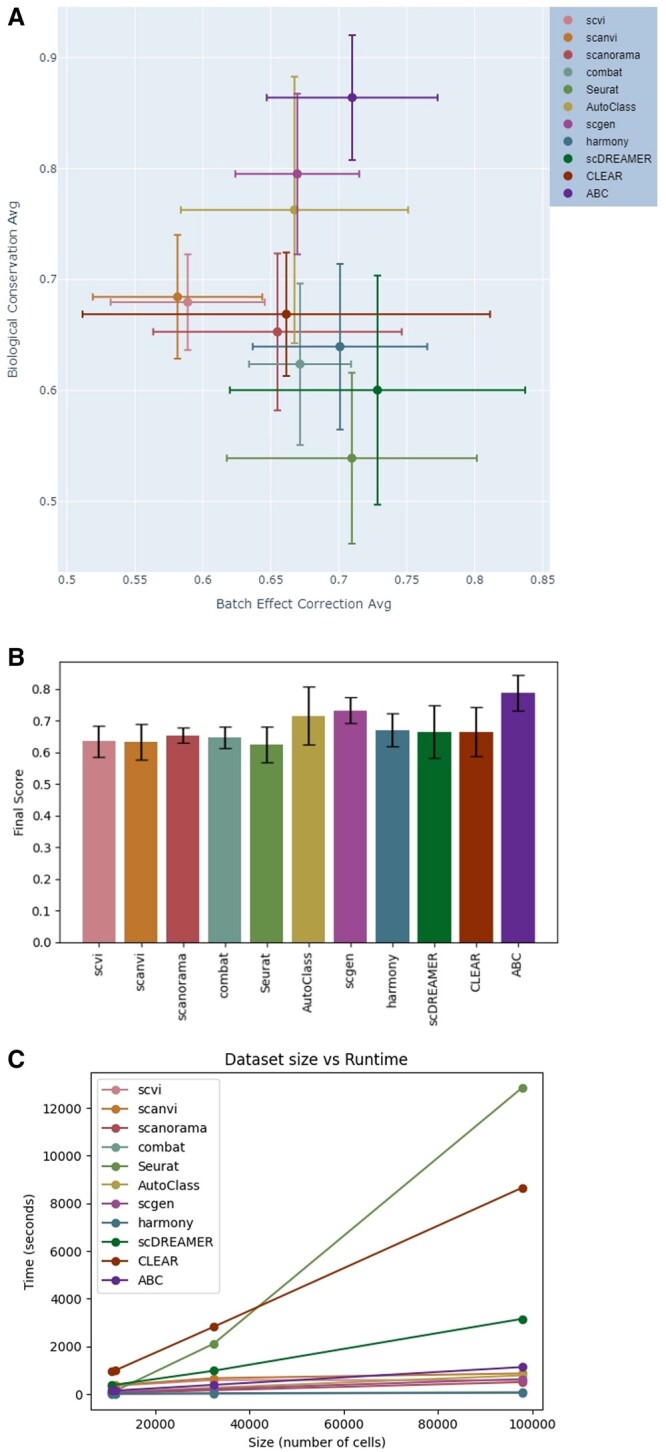
(A) Methods comparison of the trade-off between batch effects correction score and biological variance conservation score. (B) Final score comparison (ranges from 0 to 1). The score is defined as an average of the biological conservation score and the batch correction score of a method averaged over all four test datasets. (C) Scalability comparison of the different methods. Dataset size (number of cells) vs time in seconds.

We compared our results to 10 state-of-the-art batch correction methods. We defined a final score as the average of the biological conservation and batch correction scores. When comparing the average results for the four datasets we analyzed, our method’s final score outperforms all other methods (Fig. 3B). A similar result is obtained when defining the final score as a weighted average of 40% batch correction and 60% biological conservation as in Luecken *et al.* (2022).

When examining the performance in each category (batch effect removal and biological conservation), we observe that our method’s ability to conserve the biological variation is superior to other methods, while its ability to correct batch effects is better than almost all methods, except for scDREAMER, but not falling too far behind (0.71 average batch effects correction score for ABC compared to 0.728 for scDREAMER). Furthermore, our method has a very good balance between batch effect correction and biological variance conservation, compared to Seurat and scDREAMER for example, who received high batch effect correction scores but low biological variance conservation scores (Fig. 3A).

**Figure 4.**
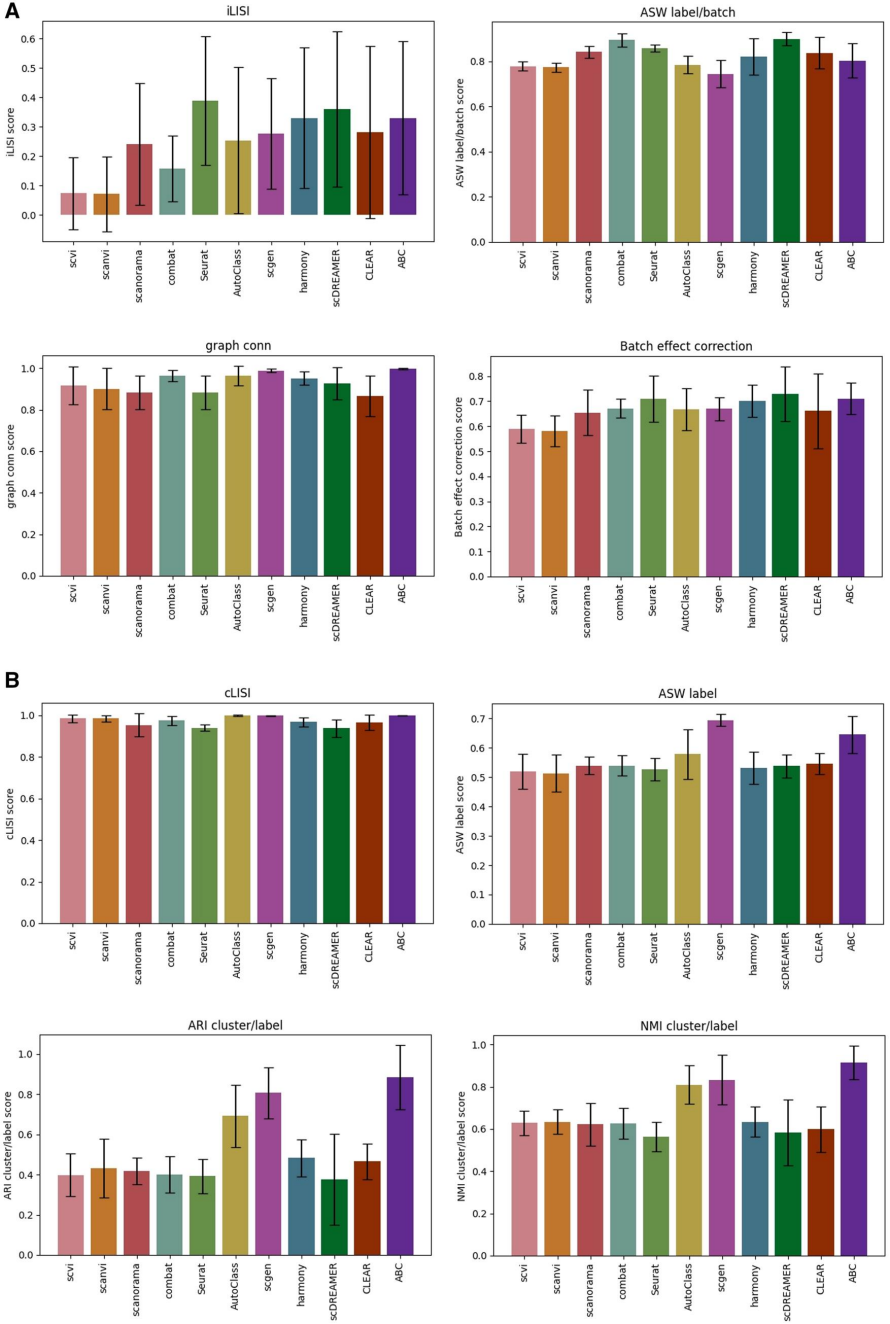
Commonly used integration metrics comparison between ABC and the various methods. (A) Batch correction metrics scores. (B) Biological variance conservation metrics scores.

A detailed comparison of batch effect correction metrics between all methods is displayed in Fig. 4A, and a comparison of four of the most commonly used biological variance conservation metrics is displayed in Fig. 4B. A comparison of all metrics can be found in Supplementary Fig. S5.

### 3.3 Preservation of spatial states in the Lung Atlas dataset

The Lung Atlas dataset includes samples from three labs, with each sample treated as a batch (lab 1: batches 1–6; lab 2: batches B1–B4 and lab 3: batches A1–A4), generated by two different techniques (Drop-seq: batches B1–B4 and 10× Chromium: batches A1–A6 and 1–6) and derived from distinct lung regions: lung parenchyma (transplant and tissue resection, batches 1–6 and B1–B4) and airway biopsies (batches A1–A6).

**Figure 5.**
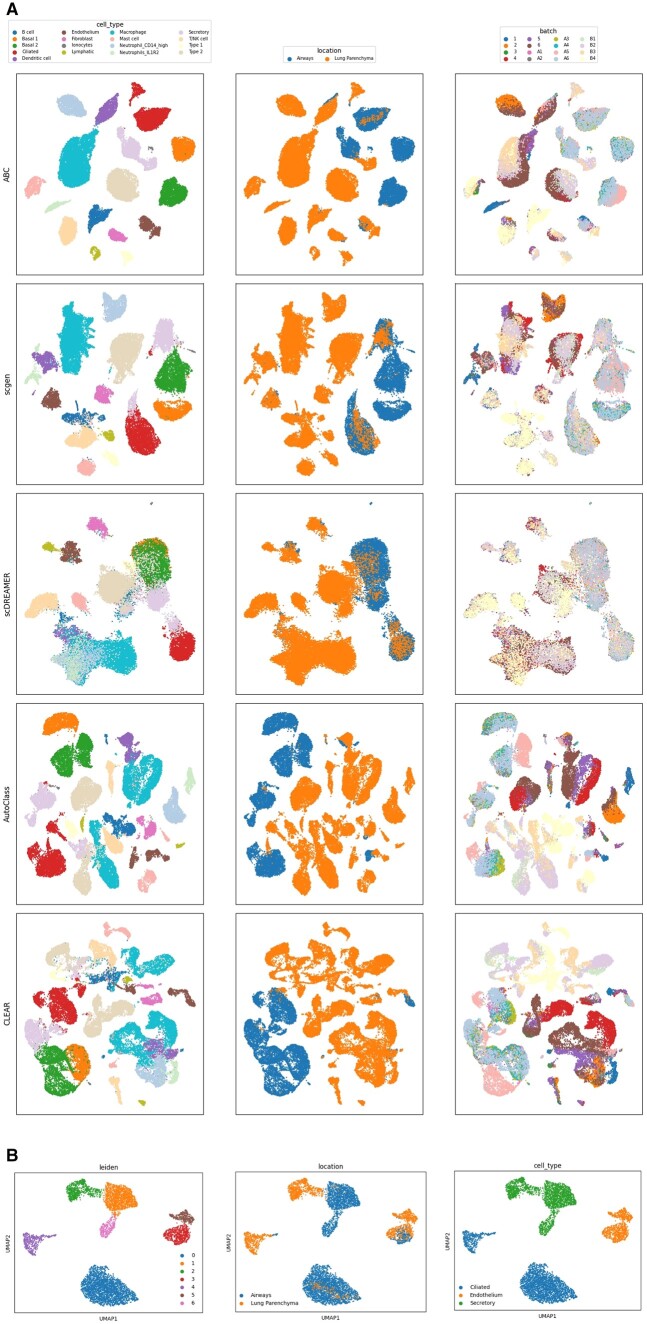
(A) Lung Atlas dataset, integrated by the various methods, colored from left to right by cell type, location, and batch labels (first five methods). (B) Lung Atlas selected cell types after integration by ABC. From left to right, colored by Leiden clustering, location and cell type. After identifying sub-clusters, there is an overlap between identified sub-clusters and spatial information in each of the selected cell types.

These different sampling locations inherently influence the cellular function and consequently the transcriptomic profiles of certain cell types. Specifically, cell types such as “Ciliated,” “Endothelium,” and “Secretory” are known to exhibit functional variations depending on their spatial context. For example, secretory cells in biopsy samples are functionally distinct from the broader category of secretory cells found in transplant samples. Similarly, endothelial cells in the lung parenchyma, involved in gas exchange, may differ functionally from those in the airway walls. Therefore, the integration of these cell types from varied locations into a single cluster may indicate a loss of biological signal, as it could obscure the functional differences inherent to their distinct origins. Our integration method, applied to this dataset, was designed to navigate these intricacies, aiming to integrate data across batch annotations while preserving the inherent biological signals. We observed that cell types like “Ciliated,” “Endothelium,” and “Secretory” displayed distinct clustering patterns between biopsy and transplant donors. The “Ciliated” cells formed two distinguishable clusters based on spatial origin, while “Endothelium” cells, although partially integrated, formed a distinct separate cluster containing cells derived from the lung parenchyma. “Secretory” cells also displayed a clear segregation between location groups (Fig. 5A). This selective integration underscores the ability of our approach to discern and maintain essential biological variation and states, attributable to the different functions and locations of these cell types, rather than indiscriminately treating this variation as mere batch effects.

Unlike other approaches, our method uniquely stands out as the only one that not only successfully integrated the different batches within the Lung Atlas but also distinctly preserved the inherent biological heterogeneity. Other methods either failed to integrate between labs or batches effectively (AutoClass, CLEAR, scANVI, scVI, and Scanorama) or, in doing so, did not preserve the biological information by mixing cell types (Harmony and Seurat), location information (scGen), or both (scDREAMER) (Fig. 5A and Supplementary Fig. S6).

### 3.4 Identification of location informed sub-populations in Lung Atlas Data

In our examination of the Lung atlas dataset, we conducted a detailed sub-clustering analysis on three major cell types—Endothelium, Ciliated, and Secretory cells (Fig. 5B), through differential expression analysis for each sub-cluster, which allowed us to discern the top differentially expressed genes and reassess the cell type annotations (Supplementary Table S1). This analysis was designed to delve deeper into the spatial information conserved post-integration, with each cell type revealing distinct sub-populations informed by their origin.

In the Ciliated cell type, Cluster 0 (blue), comprising mainly cells from airways, was marked by genes like BASP1 and CCDC78, specific to ciliated cells. The presence of these markers is consistent with the airways’ cellular composition. In Cluster 4 (purple), predominantly containing cells from Lung Parenchyma, markers of Alveolar Type 2 cells (SFTPC, SFTPB, SFTPA2, SCGB3A2) were identified. This suggests a potential reclassification from “Ciliated” to Alveolar Type 2 cells, aligning with their spatial origin. For Secretory cells, Clusters 1 (orange) and 2 (green), primarily from Airways and Lung Parenchyma respectively, showed a clear representation of Club cells and Alveolar Type 2 cells, evidenced by genes like FAM3D, MDK, and XBP1 in Club cells, and SFTPC, SFTPB, SFTPA2 in Alveolar Type 2 cells, and aligning with their origins. Cluster 6 (pink), initially categorized under the “Secretory” cells and predominantly composed of cells from the airways, unexpectedly exhibited strong expression of ciliated cell markers such as DNAAF1, LRRIQ1, RSPH1, C20orf85, and CCDC170. This finding aligns with the known abundance and functional significance of ciliated cells in the airway epithelium. It also suggests that these cells might have been originally misclassified, highlighting both the complexity of cellular phenotypes in the lung and the potential for refining cell type definitions post integration. For the Endothelium, Cluster 3 (red) exhibited a mix of cells from both Airways and Lung Parenchyma locations, primarily expressing endothelial markers such as S100A16, FXYD5 (common in Granulocytes), and VAMP5. This mixed origin underscores the diversity within the endothelial population. Cluster 5 (brown), predominantly from Lung Parenchyma, was characterized by genes indicative of Alveolar Type 2 cells, including SFTPC, SFTPA1, SFTPA2, and SFTPB, aligning with the known function of Alveolar Type 2 cells in lung parenchyma.

This fine-grained analysis aimed to further probe the spatially-informed heterogeneity following our integration method. By focusing on sub-clusters within major cell types, we discerned distinct cellular populations, each echoing their spatial origin, whether from Airways or Lung Parenchyma. These findings not only validate the precision of our integration technique but also reinforce its capability to maintain critical spatial information, essential for understanding the nuanced cellular architecture of the lung.

### 3.5 Preservation of trajectories in multi-species integration

**Figure 6.**
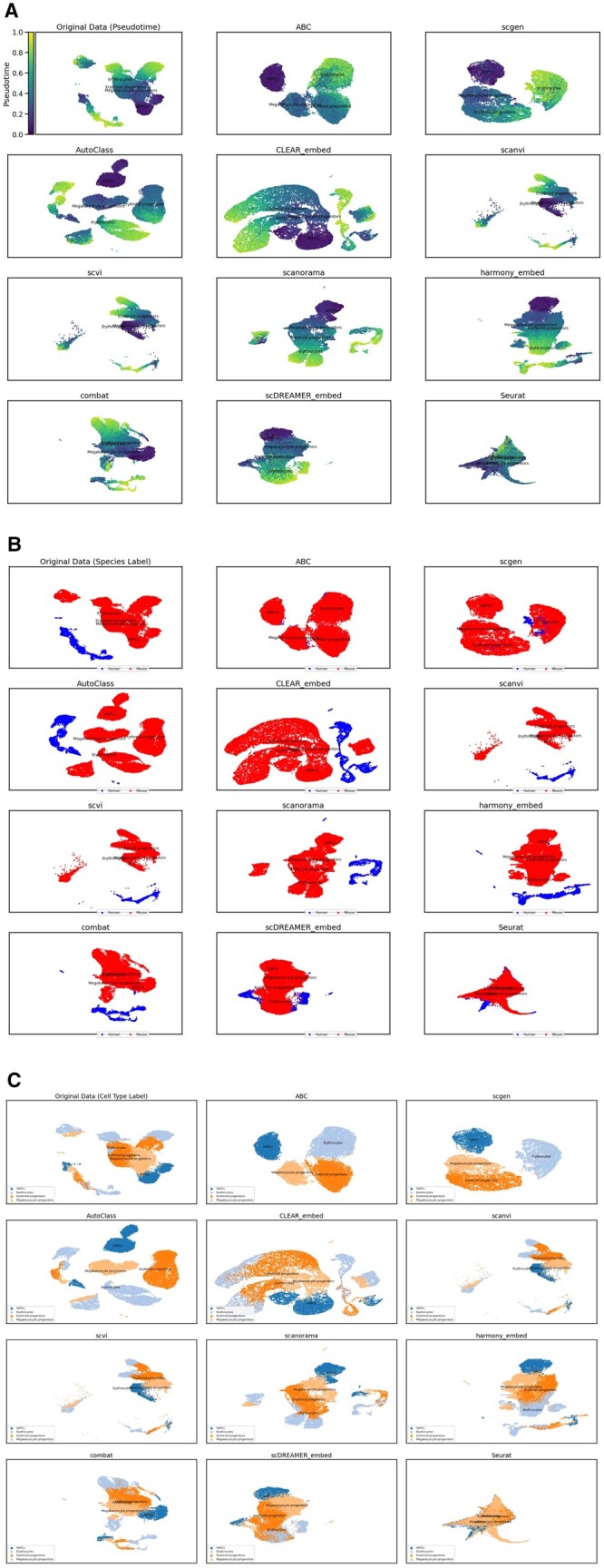
Erythropoiesis lineage cells in the Human and Mouse Immune Atlas, integrated by the various methods. (A) Cells colored by pseudotime, (B) cells colored by species, and (C) cells colored by cell types.

In our investigation, we utilized ABC’s integrated dataset of human and mouse immune cells derived from bone marrow and peripheral blood cells, focusing on erythropoiesis. The specific hematopoietic lineage we investigated included Hematopoietic Stem and Progenitor Cells (HSPCs), Megakaryocyte progenitors, Erythroid progenitors, and Erythrocytes. Employing Scanpy’s *scanpy*.*tl*.*diffmap* and *scanpy*.*tl*.*dpt* functions (Wolf *et al.* 2018), we mapped the developmental progression of these cells. Our analysis indicated that HSPCs represent the initial stage in the differentiation hierarchy. This was followed by Megakaryocyte progenitors, Erythroid progenitors and lastly Erythrocytes, characterized as the most differentiated cell type (Fig. 6A). This observation underscores the maturation path within the erythroid lineage. Overall, this pattern mirrors the general process of hematopoietic differentiation, where HSPCs give rise to various lineages, culminating in highly specialized cells like erythrocytes.

Our trajectory analysis further demonstrates the effectiveness of our integration method, successfully preserving the biological integrity of hematopoietic differentiation. The consistency of our findings with established developmental pathways (Donald 2007), underscores our method’s capability in accurately capturing the known stages of cell maturation within the immune system.

Our trajectory analysis also demonstrated a capacity for conserving global developmental trajectories across human and mouse datasets. This was particularly evident in data categorized by species label, where the data from both species exhibited a high degree of congruence in cell type progression (Fig. 6B and C). This alignment indicates a successful integration that preserves the inherent similarities in hematopoietic development between the species. These observations further demonstrate the robustness of our integration technique, highlighting its utility in maintaining a comprehensive and biologically relevant continuum of hematopoietic differentiation, regardless of species origin. Compared to other methods, ABC and scGen were the only methods able to conserve trajectories across species, with ABC displaying more defined cell type clusters.

### 3.6 Scalability

We evaluated the scalability of our integration method by examining the relationship between dataset size (number of cells) and processing time (in seconds), comparing it with other methods we tested. As illustrated in Fig. 3C, our method’s runtime demonstrates a linear increase proportional to the dataset size. Moreover, as detailed in Table 3, the runtime efficiency of our method is comparable to that of other methods, falling within the average range. Running time can be further improved by employing an early stopping approach to our architecture’s training process.

## 4 Conclusion

In this work, we developed ABC, a semi-supervised deep learning architecture based on an autoencoder. ABC integrates single**-**cell sequencing datasets and removes batch effects through a guided process of data compression, using supervised cell type classifier branches for biological signal retention. It also aligns the different batches using an unsupervised adversarial training approach. The architecture consists of an encoder, a decoder, a latent cell type classifier, and an output cell type classifier and is trained in an adversarial manner using a batch discriminator. It takes single**-**cell molecular measurements (mRNA levels or ATAC-seq levels) from a given cell and outputs a corrected vector of values.

In our study, we have effectively demonstrated that the ABC architecture is not only proficient in correcting batch effects across various datasets but also adept in preserving intricate biological variations. A key observation in all four integrated test datasets was the distinct separation of cell types and the uniform mixture of batches, highlighting the method’s ability to maintain biological integrity while addressing technical variability, in both scRNA-seq and scATAC-seq dataset.

Particularly noteworthy are the results from the Human And Mouse Immune Cells Atlas, where ABC successfully navigated the complexities of multi-species integration. It maintained the distinct identities of all 19 cell types across 23 batches, effectively mapping the intricate trajectory of hematopoietic differentiation. This included the accurate representation of developmental stages from Hematopoietic Stem and Progenitor Cells to Erythrocytes.

Furthermore, ABC was notably effective in the Lung Atlas dataset, preserving the spatial states and distinct cellular functions of sub populations. This ability to conserve spatial information, particularly in discerning functional variations of cell types like “Ciliated,” “Endothelium,” and “Secretory” cells based on their location, sets our method apart from others, who either removed these variations as batch effects or completely failed to integrate across locations or batches.

Since batch effects can have varying effects on different cell types, methods that do not account for known biological differences between cells and do not use prior knowledge as cell type labels, lack in their ability to conserve such variations and tend to remove much of the biological signal as batch effect. For example, when comparing the different methods, we can clearly see the superior biological variation conservation ability of the methods that use cell type labels (scGen, scANVI, AutoClass, and ABC).

Even though the prior knowledge of cell types can improve the integration process dramatically, this type of information is missing from many datasets, making the use of cell-type-label dependent methods like ours more difficult since it requires a complicated preprocessing step of clustering, differential expression analysis, and marker genes identification. One way to improve our method will be to add a preprocessing step to annotate a given dataset automatically. For example, we can cluster each batch separately, find differentially expressed genes and then use the overlap of these genes between clusters from different batches to match the assumed cell type clusters between batches, without identifying the cell types using known marker genes.

While a comprehensive ablation study is needed to assess the impact of each sub-module in our architecture, some insights can be gained from the performance comparison to other approaches that incorporate elements of our architecture. For instance, AutoClass employs a similar Autoencoder-based structure with a cell type classifier emanating from its latent space, akin to our method. However, it lacks the output classifier branch that aids in decompression and the discriminator for batch alignment. Another example concerns scDREAMER, which utilizes batch label information for batch alignment but does not incorporate cell type labels in the data compression and decompression process. In both comparisons, ABC demonstrated enhanced performance across various tasks underscoring the utility of each of its sub-modules.

## Supplementary Material

vbad186_Supplementary_Data

## Data Availability

All preprocessed datasets were published by Luecken *et al.* (2022) and are publicly available as Anndata objects on Figshare at: https://doi.org/10.6084/m9.figshare.12420968. ABC is freely available on github at https://github.com/reutd/ABC.
