# Supplementary material for: Batch correction of single-cell sequencing data via an autoencoder architecture
Source: Bioinform Adv. 2023 Dec 28;4(1):vbad186. doi: 10.1093/bioadv/vbad186 (PMC10781938; doi:10.1093/bioadv/vbad186)
Supplement: vbad186_Supplementary_Data [file vbad186_supplementary_data.pdf]

Batch correction of single cell sequencing data via an autoencoder architecture - Supplementary Information

Reut Danino[1], Iftach Nachman[2], Roded Sharan[1,*]

[1] Blavatnik School of Computer Science, Tel Aviv University, Tel Aviv, 6997801, Israel
[2] School of Neurobiology, Biochemistry and Biophysics, George S. Wise Faculty of Life Sciences, Tel Aviv University, Tel Aviv, 6997801, Israel

*Corresponding author: Roded Sharan, roded@tauex.tau.ac.il

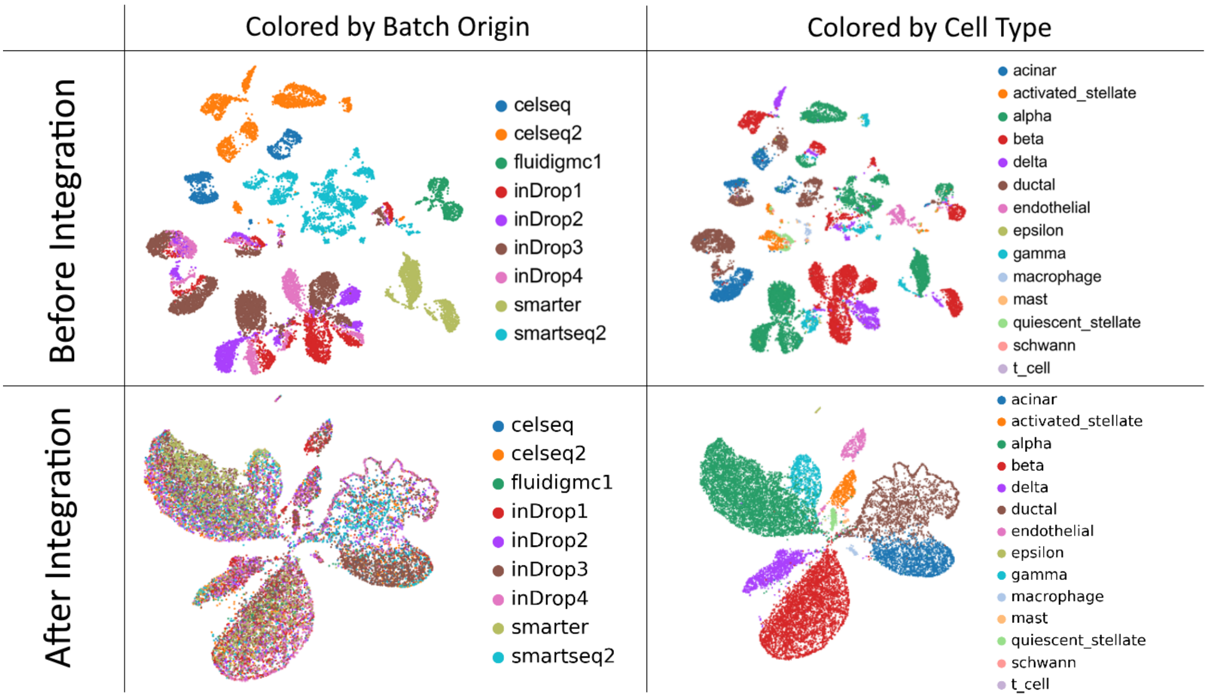

Figure S1: Human Pancreas dataset projected on a UMAP, before and after integration by ABC. This dataset contains 16,382 cells from 9 different sources (batches) and 14 cell types.

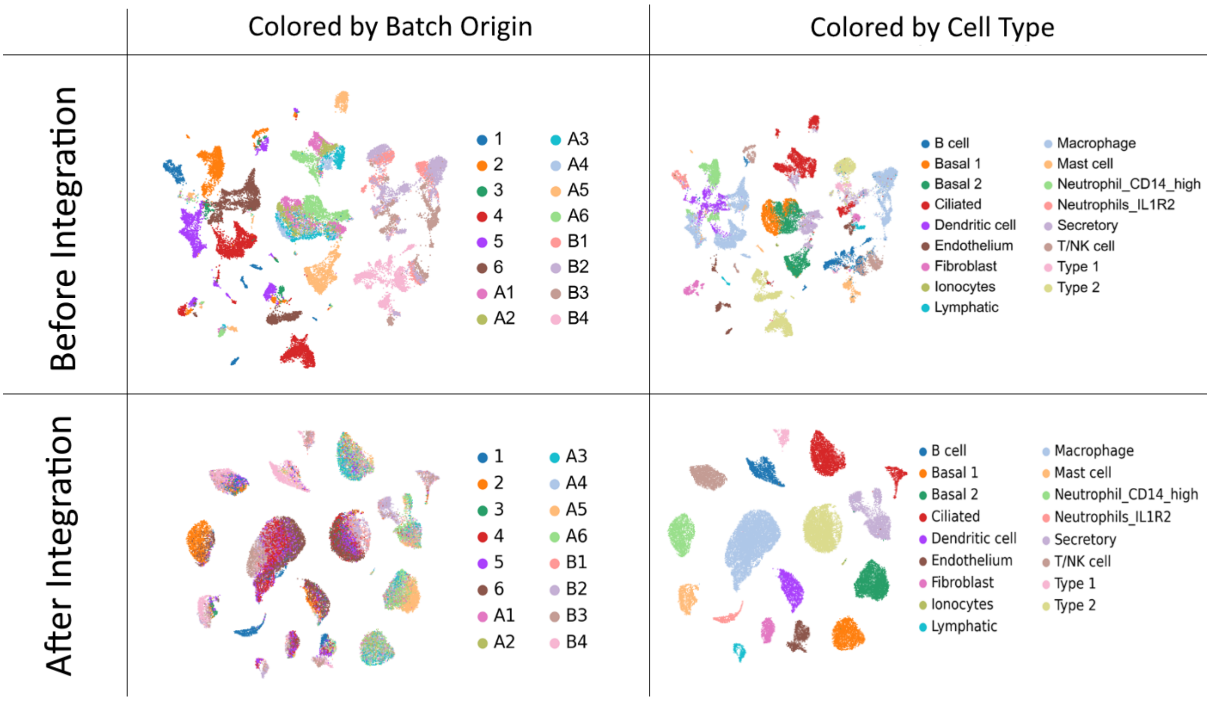

Figure S2: Human Lung Atlas dataset projected on a UMAP, before and after integration by our method. This dataset contains 32,472 cells from 16 different sources (batches) and 17 cell types.

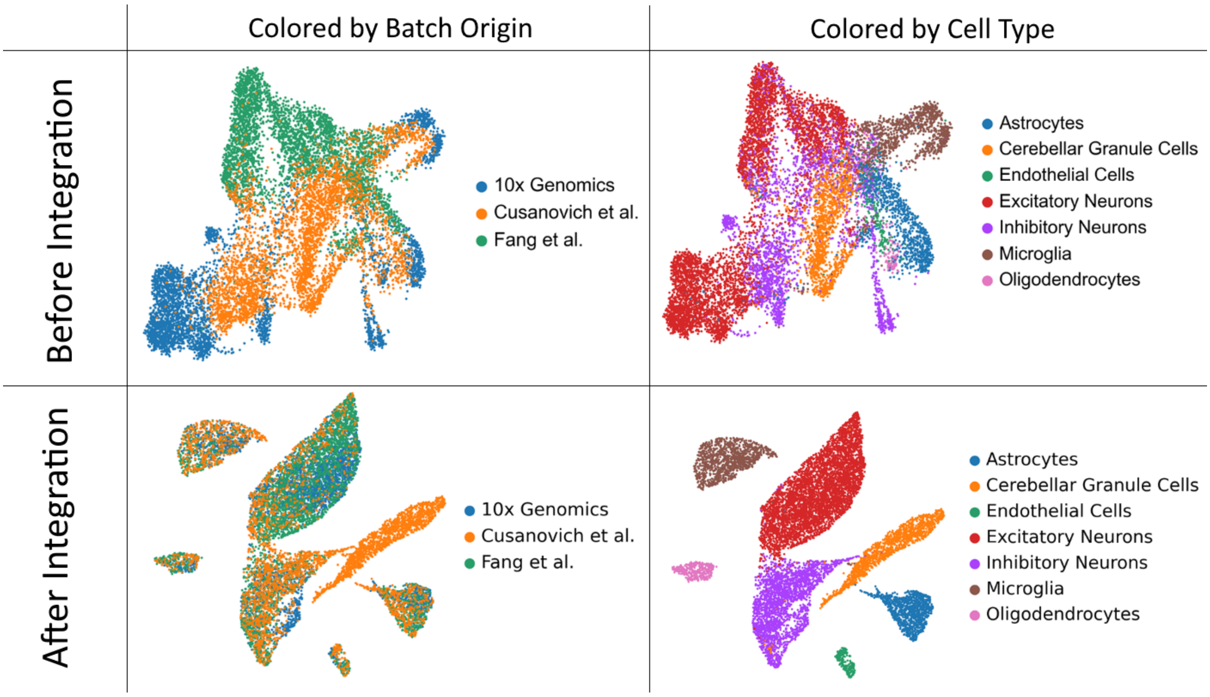

Figure S3: Small mouse brain (ATAC) Gene Activity dataset projected on a UMAP, before and after integration by ABC. This dataset contains 11,270 cells from 3 different sources (batches) and 7 cell types.

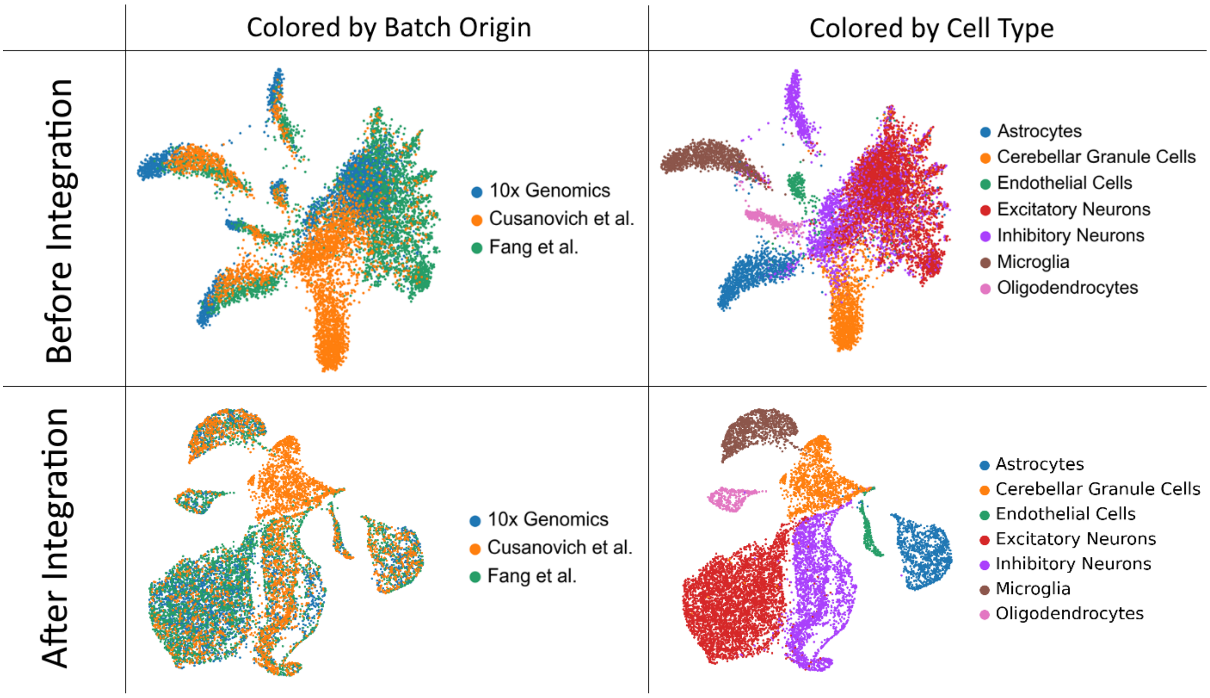

Figure S4: Small mouse brain (ATAC) Windows dataset projected on a UMAP, before and after integration by ABC. This dataset contains 10,761 cells from 3 different sources (batches) and 7 cell types.

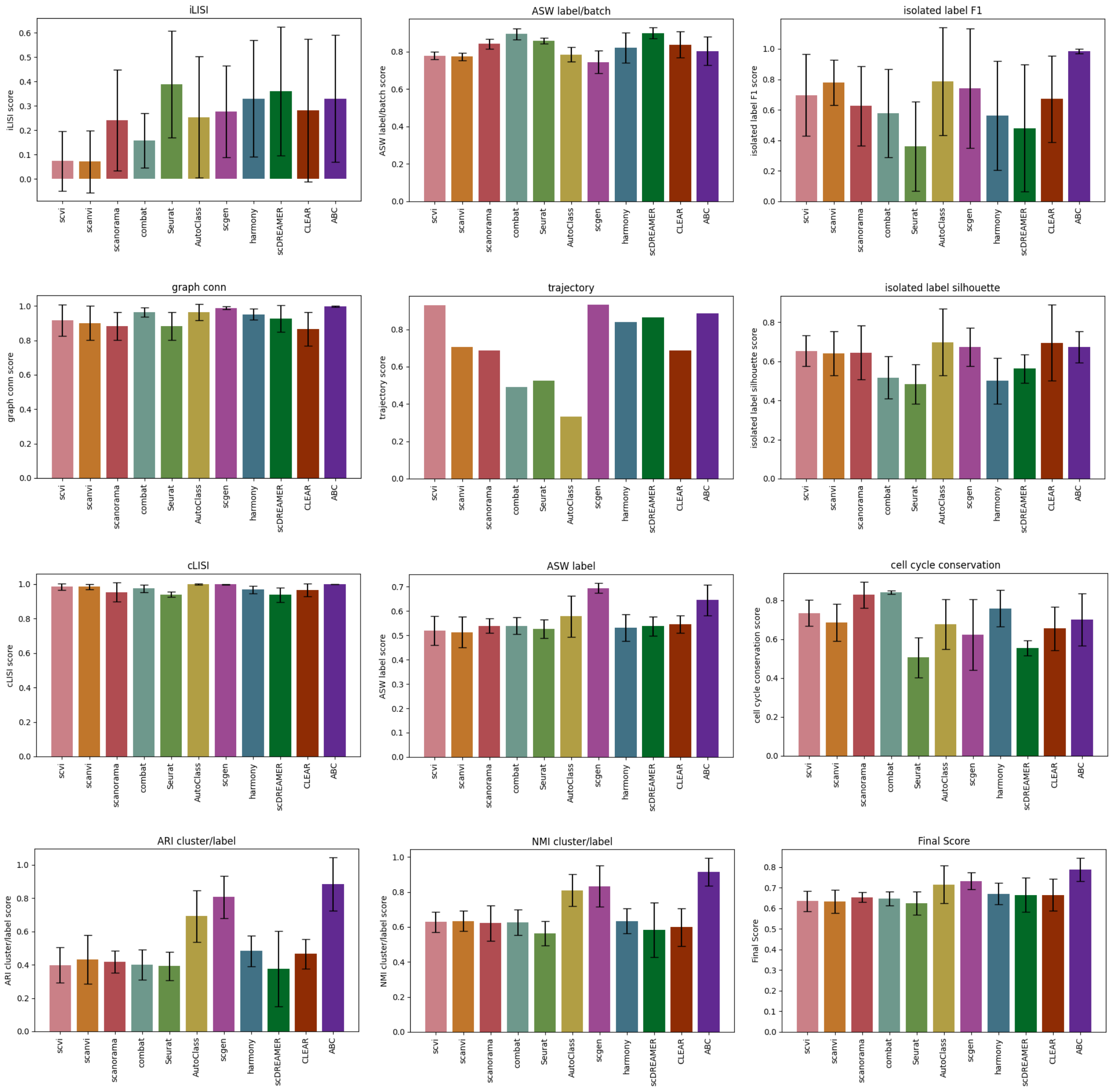

Figure S5: All metrics comparison of methods. Three batch effect removal metrics were used: ASW (batch), Graph Connectivity and Graph iLISI, and eight biological variance conservation metrics were used: NMI, ARI, ASW (cell-type), Graph cLISI, Isolated label F1 and Isolated label silhouette, Cell cycle conservation and Trajectory conservation.

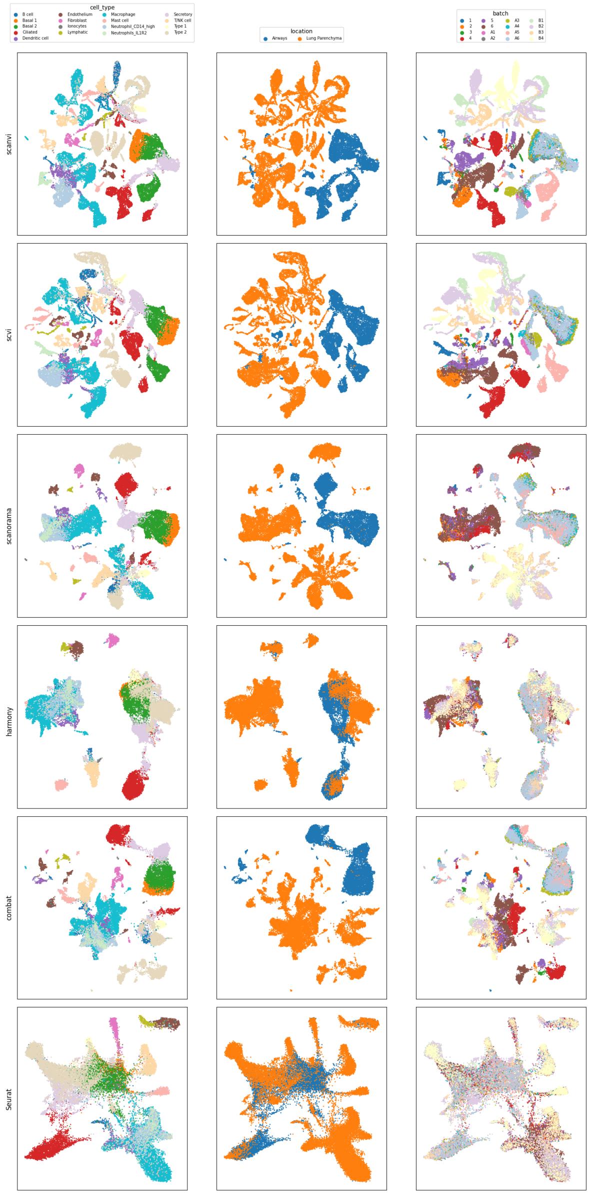

Figure S6: Lung Atlas dataset, integrated by the various methods, colored from left to right by bell type, location and batch labels (last six methods).

Table S1: Differential Expression in Lung Atlas Sub clusters

Clusters DE Genes	Scores	Associated Cell Type
Endothelium Cluster 3		
S100A16	34.3	Endothelial cells
FXYD5	29.1	Granulocytes
CCDC85B	28.2	Endothelial cells
PRCP	27.8	Endothelial cells
VAMP5	26.9	Endothelial cells
Endothelium Cluster 5		
SCGB1A1	22.2	Club cells
SFTPC	21.3	Alveolar cells type 2
SFTPA1	11.9	Alveolar cells type 2
SFTPA2	11.7	Alveolar cells type 2
SFTPB	10.3	Alveolar cells type 2
Ciliated Cluster 0		
BASP1	69.6	Ciliated cells
CCDC78	68.1	Ciliated cells
TCTEX1D4	67.0	Ciliated cells
IGFBP2	66.9	Ciliated cells
DYX1C1	65.1	Ciliated cells
Ciliated Cluster 4		
SFTPC	21.4	Alveolar cells type 2
SCGB1A1	15.7	Club cells
SFTPB	14.7	Alveolar cells type 2
SFTPA2	12.9	Alveolar cells type 2
SCGB3A2	12.1	Alveolar cells type 2
Secretory Cluster 1		
FAM3D	62.2	Club cells
MDK	59.4	Club cells
TSPAN8	58.2	Club cells
XBP1	56.7	Club cells
VMO1	54.1	Club cells
Secretory Cluster 2		
SFTPC	42.1	Alveolar cells type 2
SFTPB	36.5	Alveolar cells type 2
SFTPA2	30.2	Alveolar cells type 2
SCGB3A2	27.6	Alveolar cells type 2
SFTPA1	24.8	Alveolar cells type 2
Secretory Cluster 6		
DNAAF1	66.5	Ciliated cells
LRRIQ1	60.3	Ciliated cells
RSPH1	59.4	Ciliated cells
C20orf85	55.6	Ciliated cells
CCDC170	53.7	Ciliated cells
